# Utilization of Ethylcellulose Microparticles with Rupatadine Fumarate in Designing Orodispersible Minitablets with Taste Masking Effect

**DOI:** 10.3390/ma13122715

**Published:** 2020-06-15

**Authors:** Katarzyna Wasilewska, Patrycja Ciosek-Skibińska, Joanna Lenik, Stanko Srčič, Anna Basa, Katarzyna Winnicka

**Affiliations:** 1 Department of Pharmaceutical Technology, Medical University of Białystok, Mickiewicza 2c, 15-222 Białystok, Poland; katarzyna.wasilewska@umb.edu.pl; 2 Chair of Medical Biotechnology, Warsaw University of Technology, Noakowskiego 3, 00-664 Warsaw, Poland; pciosek@ch.pw.edu.pl; 3 Department of Analytical Chemistry and Instrumental Analysis, Faculty of Chemistry, Maria Curie-Skłodowska University, M. Curie-Skłodowska Sq. 3, 20-031 Lublin, Poland; j.lenik@poczta.umcs.lublin.pl; 4 Department of Pharmaceutical Technology, University of Ljubljana, Aškerčeva c. 7, 1000 Ljubljana, Slovenia; stanko.srcic@ffa.uni-lj.si; 5 Department of Physical Chemistry, Faculty of Chemistry, University of Białystok, Ciołkowskiego 1K, 15-245 Białystok, Poland; abasa@uwb.edu.pl

**Keywords:** ethylcellulose, spray drying, microparticles, rupatadine fumarate, orodispersible minitablets, taste masking

## Abstract

Minitablets in orodispersible form constitute a flexible drug delivery tool for paediatric and geriatric population as they eliminate the risk of chocking and do not require drinking water in the application. Due to their direct contact with taste buds, taste sensation is an important factor. Preparing microparticles with taste masking polymers utilizing spray drying is an efficient technique for reducing the bitterness of drugs. Ethylcellulose is a hydrophobic polymer widely used as a taste masking material. Rupatadine fumarate, one of the newest antihistamines, features an intensive bitter taste, hence in designing orodispersible formulations, achieving an acceptable taste is a crucial issue. The main objective of this work was to formulate orodispersible minitablets containing taste masked ethylcellulose-based microparticles with rupatadine fumarate and evaluation of their quality, especially in terms of taste masking efficacy. The accessed data indicated that all obtained minitablets were characterized by beneficial pharmaceutical properties. Three independent methods: in vivo with healthy volunteers, in vitro drug dissolution, and “electronic tongue” confirmed that all designed formulations provided satisfactory taste masking rate and that formulation F15 (prepared with Pearlitol^®^ Flash and Surelease^®^ microparticles with rupatadine fumarate) was characterized by the lowest bitterness score.

## 1. Introduction

The challenge of modern pharmaceutical technology is designing easy-to-administer drug dosage forms where the dose is sufficiently flexible to enable proper application and dose titration both to paediatric and adult patients. The currently available formulations that might be used in any age population are primarily liquids. However, the barrier of their utilization are difficulties in effective taste masking of bitter active pharmaceutical ingredients (API), the necessity of applying a large volume of medical preparation to adults, as well as low physicochemical and microbiological stability. Therefore, new technological solutions and strategies are being sought, as the appropriate drug formulation should be acceptable for a wide age group, in terms of organoleptic properties (taste, smell, appearance) to ensure regular intake of medicine, even while prolonged therapy. In case of solids, a crucial element that decides if they are swallowed, is their size—the smaller the unit, the easier application [1,2,3,4].

The solid drug dosage form, which connects the advantages of liquids (flexibility of dosing, ease of swallowing) with the qualities of solids (taste masking, stability), as well as enables individual dose adjustments for patients of all ages are minitablets (MT). MT are created for those encountering difficulties with application of larger tablets or as a form providing the possibility of dose titration by “multiplication”, which allows the use of one product for the entire age population. The appropriate dose is determined by the number of MT administered (e.g., children of different ages will take different number of MT as one dose). MT are characterized by small sizes of one to three millimeters and mass of 5–25 mg. They are produced like traditional tablets, using existing technologies and production lines, as well as standard tableting blends. A promising type of MT are orodispersible minitablets (ODMT), which are characterized by very short disintegration and dissolution rates. ODMT are particularly recommended for patients with swallowing problems, by eliminating the risk of choking to a minimal [5,6,7,8,9,10,11].

The major limitation in designing orodispersible formulations is unpleasant taste of API. Insufficient taste masking effect of medicine is the most common reason for refusing of taking the preparation [12]. Taste masking techniques can be divided in two main groups: chemical modifications of API to reduce its solubility or creating a physical barrier between drug molecules and taste receptors. The first group includes primarily conversion into a prodrug (ester, salt), complexation with cyclodextrins, or ion exchange resins. The second mechanism involves designing microparticles or coatings using polymers having limited solubility in the oral cavity environment. A useful method of decreasing unsavory taste of a medicine is obtaining microparticles utilizing taste masking polymers. There are several technologies for preparing microparticles, among which spray drying is one of the most effective and efficient [12,13,14,15,16].

The aim of the following paper was to create ODMT containing microparticles with rupatadine fumarate (RUP) as a model bitter drug. Microparticles were obtained employing the spray-drying, utilizing ethylcellulose as a barrier forming polymer. In our previous work, microparticles prepared using different forms of EC were compared. The microparticles prepared with an aqueous dispersion of EC were found to have better properties in terms of taste masking effectiveness and morphology [17], therefore they were used to formulate ODMT. ODMT were evaluated regarding their morphological structure utilizing scanning electron microscopy (SEM), uniformity of weight and thickness, mechanical properties, drug content, and potential interactions occurring using differential scanning calorymetry. Disintegration time was evaluated in vivo by healthy volunteers on petri dishes and with texture analyzer usage. The crucial test—assessment of taste masking effect was carried out according to three alternative approaches: in vivo, by the drug dissolution and with electronic tongue utilization.

## 2. Materials and Methods

### 2.1. Materials

Aquacoat^®^ ECD was donated from FMC BioPolymer, Newark, NJ, USA. Surelease^®^ E-7-19040 was given from Colorcon Inc., Harleysville, PA, USA. Parteck^®^ ODT was received from Merck KGaA, Darmstadt, Germany. SmartEx^®^ QD-50 was handed over from Shin-Etsu Chemical Co., Ltd., Tokyo, Japan. F-Melt C was purchased from Fuji Chemical Industry Co., Ltd., Toyama, Japan. Pearlitol^®^ Flash was a gift from Roquette, Lestrem, France. Magnesium stearate and methylene blue were acquired from POCh, Piekary Śląskie, Poland. RUP was procured from Xi’An Kerui Biotechnology Co., Ltd., Xi’An, China.

### 2.2. Preparation of ODMT

A traditional tablet presser (Type XP1, Korsch, Berlin, Germany) with 3-mm punches was employed to manufacture ODMT by direct compression. In tableting bulk preparation, optimized spray dried microparticles with RUP prepared with EC aqueous dispersions selected during preliminary studies—urelease^®^ and Aquacoat^®^ ECD as a barrier coatings were utilized [17]. The conditions of the spray drying process were established experimentally: 85 °C, aspirator flow 98%, rate of flow 3.5 mL/min. Efficient barrier for masking the bitterness of drug enclosed in microparticles was obtained utilizing RUP:polymer ratio (0.5:1) with 6% EC concentration and this formulation was used for designing ODMT. ODMT with a mass of 14 mg and amount of microparticles corresponding to 0.5 mg of RUP per single tablet were assumed. The compositions of designed tableting masses utilized in the study are shown in Table 1. Prepared tableting blends were mixed manually for 30 s. To determine relevant conditions of the tableting process, various pressure force grades ranging from 0.6 to 1.2 kN were tested. Tablets with optimal properties that did not have a damaged surface of microparticles were obtained using a 0.9 kN (± 0.1) force. To simplify the formulation of ODMT, multifunctional co-processed mixtures were utilized. Co-processed mixtures are designed by processing several excipients to possess advantages that cannot be achieved by the simple physical mixtures of their components [18,19,20,21,22,23,24].

### 2.3. Flow Properties of Powders

The tableting blends (Table 1) were subjected to preformulative quality assessment in accordance with pharmacopoeial requirements [25]. Each study was carried out in triplicate. A tapping apparatus (Electrolab ETD-1020, Mumbai, India) was utilized for the compressibility studies. The bulk and tapped densities were calculated as quotients of the weight of the powder to its volumes occupied before and after tapping and then the powder density index (Index Carr) and the powder flow index (Hausner’s ratio) were calculated. To investigate powder flow time, 50 g of the sample was placed in the funnel with the outlet closed and after opening the valve, the flow of the whole sample through the funnel was measured [25].

### 2.4. Evaluation of Morphology of ODMT

Morphological structure was assessed utilizing scanning electron microscopy (Inspect™S50, FEI Company, Hillsboro, OR, USA). Swatches were placed on adhesive tapes fixed to the surface of a special stand and gold sprayed. Tests were performed at room temperature, using various magnifications.

### 2.5. Quality Assessment of ODMT

#### 2.5.1. Uniformity of Weight and Thickness

Twenty randomly chosen ODMT were weighted individually, employing analytical balance (Radwag, Radom, Poland) [25]. The thickness was tested with calibrated digital caliper utilization (Beta 1651DGT, Milan, Italy).

#### 2.5.2. Mechanical Properties

Tests were conducted with friability tester (EF-1 W, Electrolab, Mumbai, India) according to pharmacopoeial monograph for a quantity of ODMT corresponding to 6.5 g [25]. The crushing strength of ODMT was tested utilizing a hardness tester (5Y, Pharmaton AG, Thun, Switzerland) and a Texture Analyzer TA.XT. Plus (Stable Microsystems, Godalming, UK) with a steel cylinder of 6 mm diameter and 0.1 mm/s pre-speed [25]. The minimum force (N) needed to crush the ODMT was measured by vertically applying pressure along its diameter. Ten randomly selected tablets from the batch were used for hardness assessment in both methods.

#### 2.5.3. Drug Content

HPLC apparatus (Agilent Technologies 1200) equipped with Waters Spherisorb^®^ 5 μm ODS1 4.6 × 250 mm column (Waters Corporation, Milford, CT, USA) was applied to evaluate RUP content uniformity for individual ODMT. As a mobile phase methanol:phosphate buffer pH = 3.0 (35:65, v/v) was utilized (isocratic elution). Flux was established as 1.0 mL/min and wavelength as 245 nm. Buffer was composed of NaH_2_PO_4_ and water, adjusted to pH = 3.0 by H_3_PO_4_. Standard calibration curve was linear in the range of 1–100 μg/mL and the correlation coefficient R^2^ was 0.999. The studies were carried out in triplicate [26,27,28].

### 2.6. Disintegration Time Assessment

#### 2.6.1. In Vivo

The study was carried out by six probands (Research Ethics Committee at Medical University of Białystok approval number R-I-002/438/2016) in the following stages: oral cavity rinsing with water, placing ODMT in the mouth without chewing until disintegration, splitting out. The time needed for the total disintegration in the mouth was recorded.

#### 2.6.2. Petri Dish

Petri dish having a diameter of 7 cm was filled with 4 mL of phosphate buffer pH = 6.8 imitating natural spit (composing of Na_2_HPO_4_; KH_2_PO_4_ and water; adjusted to pH 6.8 by 1-M NaOH) [17] and single ODMT was put in the center. The test was repeated for 6 tablets from each batch. Time for the tablet to completely disintegrate into fine particles was measured.

#### 2.6.3. Texture Analyzer

**Figure 1:**
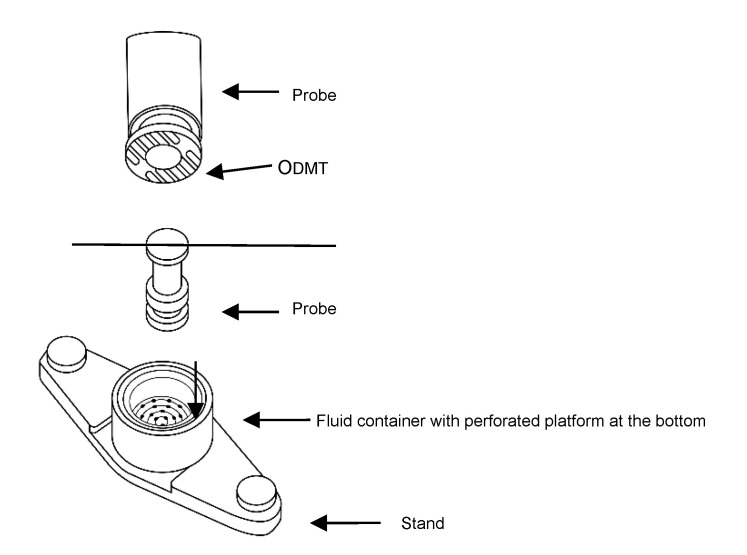
Schematic illustration of disintegration time rig of the texture analyzer.

The study was conducted with a texture analyzer (Stable Microsystems, Godalming, UK) with an ODT disintegration time rig (Figure 1). Single ODMT was attached to the bottom of the probe with double-side adhesive tape. The ODMT was immersed in the medium (4 mL of phosphate buffer pH = 6.8 imitating natural spit) until it comes in contact with the perforated bottom of the container. The test was repeated for 6 tablets from each batch.

#### 2.6.4. Wettability

The wettability test was carried out according to Stoltenberg and Breitkreutz [29]. A 96-well plate (Biologix Group Limited, Lenexa, KS, USA) was used and a cellulose filter disc was placed in each well, which was then moistened by adding 20 µL of 0.3% aqueous solution of methylene blue. The time from placing the ODMT on moistened paper to complete coloring of the MT matrix was noted.

#### 2.6.5. Differential Scanning Calorimetry

RUP raw material (API), microparticles placebo (MP AQ placebo, MP SUR placebo), microparticles (MP AQ RUP, MP SUR RUP), ODMT placebo (F1, F5, F9, F13), ODMT with pure RUP (F2, F6, F10, F14) and ODMT with RUP enclosed in SUR MP (F3, F7, F11, F15) and AQ MP (F4, F8, F12, F16) were tested using thermal analyzer system (DSC Mettler Toledo, Greifensee, Switzerland). Swatches were accurately weighed (5 mg), inserted in pans made of aluminum, then warmed up to 300 °C with 10 °C/min rate with 20 mL/min flow of nitrogen.

### 2.7. Evaluation of Taste Masking Effectiveness

#### 2.7.1. In Vivo

The study was conducted in accordance with the Declaration of Helsinki and the protocol was approved by the Ethics Committee of Medical University of Białystok approval number R-I-002/438/2016. The efficiency of taste masking level was tested by six probands undergoing a test conducted as follows: five ODMTs were placed in the oral cavity for 30 s (the maximum time to dissolve/disintegrate in accordance to FDA guidelines [30]), spitted and mouth were rinsed with water. Sensory evaluation was marked as follows: 0—no bitterness, 1—slightly bitterness, 2—moderately bitterness, 3—significant bitterness. Before the experiment was carried out, the participants were chosen on the basis of sensory sensitivity test, utilizing four main flavors [31].

#### 2.7.2. RUP Dissolution

RUP dissolution was carried out in apparatus II (paddle) (Erweka Dissolution Tester DT 600HH, Heusenstamm, Germany) with phosphate buffer (pH 6.8, 50 mL) imitating natural spit in following conditions: 75 rpm and 37 °C (+/−0.5). The quantity of dissolved RUP was assessed as pointed in 2.5.3.

#### 2.7.3. Electronic Tongue

##### Reagents and Membrane Materials

Analytical reagent grade chemicals and purified water with 0.07 μS/cm conductivity (Elix Advantage System Mili-Q plus Milipore, Spittal an der Drau, Austria) were used. The membrane consisted of poly(vinylchloride) (PVC) (Tarwinyl, Tarnów, Poland); bis(2-ethylhexyl) sebacate (DOS), and o-nitrophenyl octyl ether (o-NPOE) (Fluka, St. Gallen, Switzerland); potassium tetrakis [3,5-bis(trifluoromethyl)phenyl]-borate (KTFPB), tridodecylmethylammonium chloride (TDMAC), 1-dodecylpyridinium chloride (DDPC), (Sigma—Aldrich, St. Luis, MO, USA), and potassium tetrakis(p-chlorophenyl)borate (KTpCPB) (Fluka, St. Gallen, Switzerland); calix[6]arene-hexaacetic acid hexaethylester (amine ionophore I) (Fluka, St. Gallen, Switzerland), and 3-mercapto-5-/2′-hydroxynaphthyl-azo-triazole (METRIAN) (Department of Drug Chemistry, Medical University of Lublin, Poland).

##### Membrane Preparation

Each solid contact electrode consists of a conventional body and an exchange Teflon holder in which the two phases are placed. The interior lamina (1) contains PVC with plasticizer in which the Ag/AgCl electrode is inserted and the exterior lamina (2) contains the ion-sensitive component and inner layer components. The exterior lamina is in contact with the tested solutions. The steps of the membrane phase preparation:

(1) weighing inner layer components: 30% (*w/w*) PVC, 70% (*w/w*) of plasticizers, DOS or o-NPOE,
(2) mixing and deaerating of obtained mixture,
(3) filling the Teflon holder with mixture to cover the silver-silver chloride electrode,
(4) gelating inner layer (1) at 373 K for 30 min, cooling of the gelled layer,
(5) weighing outer layer components, 27–33% (*w/w*) of PVC, 64–68% (*w/w*) of plasticizers, 1–5% of electroactive components (2) (Table 2),
(6) dissolving of obtained mixture in THF,
(7) placing drops on the inner layer (1),
(8) gelating outer layer (2) in result of evaporation THF at 293 K; repeating the steps several times.

##### Potentiometric Measurements

EMF electrochemistry interface system (Lawson Labs. Inc., Malvern, PA, USA) and IBM PC were used for potentiometric measurements. The potentiometric sensor array of the system contains 16 solid contact ion-selective electrodes (two sensors of each type) differing active substances and plasticizers each other. The constructed solid contact electrodes were stored for 24 h in RUP solutions before the first measurement. As a standard, Ag/AgCl electrode (Orion 90-02, Thermo Electron Corporation, Beverly, MA, USA) was used. The electrodes calibration curves were performed in 10^−5^–10^−3^ mol L^−1^ RUP solutions because of the poor solubility of RUP in water. Previously developed measurement protocol for electronic tongue analysis of pharmaceutical samples [32] was applied to test taste masking and API dissolution from designed ODMTs. First, the sensors were immersed in deionized water (50 mL) to obtain 5 min signal stabilization. Then adequate samples were added and released API and excipients influenced electrodes’ signals, that were recorded as the changes of potentials (ΔEMF) of particular electrodes in sensor array in a function of time. The release measurements were carried out for solutions of pure API (RUP), all studied ODMTs and respective placebos. The signals of the sensors were registered during 15 min (5 min stabilization, 10 min release), in 5 repetition for each sample type. The sensors were water rinsed and dried between assays.

##### Data Analysis

Data matrix was composed of data vectors assigned to every investigated formulation (16 variables for every time-point, responding to 16 ΔEMF signals of 16 potentiometric sensors). The data matrixes were processed by chemometric procedures: Principal Component Analysis (PCA) or Partial Least Squares (PLS). These calculations as well as data analysis and presentation were performed in SOLO^®^ software (Eigenvector Research Inc., Manson, WA, USA).

## 3. Results and Discussion

### 3.1. Pharmaceutical Evaluation of ODMT

**Figure 2:**
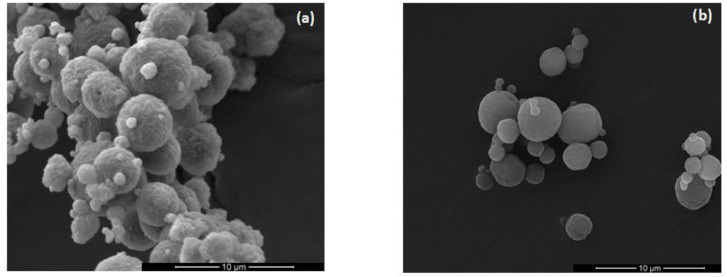
SEM picture of microparticles prepared using: (**a**) Surelease^®^, (**b**) Aquacoat^®^ ECD under magnification 10,000×.

An attempt was made to design and develop ODMT as an innovative drug dosage form, utilizing RUP enclosed in ethylcellulose microparticles to reduce bitterness. In preparation of ODMT, mainly spherical, homogenous, smooth surfaced microparticles based on EC aqueous dispersions were used (Figure 2). The mean size of microparticles made from Surelease^®^ was 3.2 +/− 1.1 µm and from Aquacoat^®^ ECD was 3.6 +/− 1.5 µm

As a model drug with bitter taste, RUP (a long-acting second generation antihistamine showing anti-allergic and demulcent effect applied both in children and adults) was utilized. RUP is the histamine receptor selective antagonist and receptor for platelet activating factor (PAF), which highlights it from drugs belonging to this group and clarifies its unique mechanism of action. RUP binds to the H_1_ receptor permanently and firmly, acting as an inverse agonist, which prolongs duration of its action. It was indicated that RUP is characterized by far greater affinity to H_1_ receptor than fexofenadine or levocetirizine. Furthermore, by binding to PAF receptors, RUP causes their blockade, what is clinically relevant to PAF allergic inflammatory processes and bronchial hyperreactivity symptoms. RUP has not only been shown to reduce the amount of erythema (which is characteristic of all antihistamines), but also reduces PAF-induced platelet aggregation. The third component of RUP activity is its additional anti-inflammatory effect consisting in: inhibition of mast cell degranulation and release of histamine and cytokines (e.g., IL-4,5,6,8, TNF α), inhibition of eosinophil and neutrophil chemotaxis, inhibition of expression of adhesive molecules (CD18, CD11b) and transcription factors [33,34,35,36,37,38,39]. Commercially it is available in traditional tablets form [40] and due to its unpleasant taste, there is no orodispersible drug dosage forms on the pharmaceutical market.

To reduce bitterness of RUP, ethylcellulose (EC), a hydrophobic polymeric material widespread applied in masking the unpleasant aroma and taste was applied. It belongs to the GRAS (generally regarded as safe) and FDA Inactive Ingredients [41]. Moreover, EC is considered not to carry any health risks, therefore its daily intake has not been explicated by the World Health Organization (WHO) [42]. It is an ethyl ether of cellulose, in the form of a free-flowing, odorless, tasteless, biocompatible, non-allergenic, and nonirritant white to light-tan powder dissolving only in organic media, thus creating a polymeric barrier that allows for temporary isolation of a bitter drug from the oral cavity environment [43,44,45]. EC is accepted to be utilized in paediatric medicinal products, as well as in non-parenteral formulations authorized in Europe [41,46,47]. EC is available in organic form (e.g., Ethocel^®^) and as aqueous dispersions (e.g., Surelease^®^, Aquacoat ECD^®^). Surelease^®^ contains a 25% of solid EC and dibutyl sebacate and oleic acid as plasticizers. In Aquacoat^®^ ECD there is 27% EC, sodium lauryl sulfate, and cetyl alcohol. The dispersions are accepted for pharmaceutical use in the Europe, United States, and Japan [48,49].

ODMT were prepared by direct compression method, using commercially available mixtures: Parteck^®^ ODT, SmartEx^®^ QD-50, FMelt^®^ C, Pearlitol^®^ Flash. The amount of API was set as 0.5 mg per one ODMT. The dose selection was related to the fact that by multiplication, the dose of 2.5 mg required for children weighting from 10 to 25 kg can be easily achieved. No significant technological problems were observed during tableting process. It was connected with low API content in the tablet masses, so it did not affect the flowing properties of powders. The similar composition of all mixtures based mainly on mannitol caused all the obtained formulations to be characterized by similar physical parameters (Table 3). The best flowability was noted for blends with FMelt^®^ C and Pearlitol^®^ Flash.

**Figure 3:**
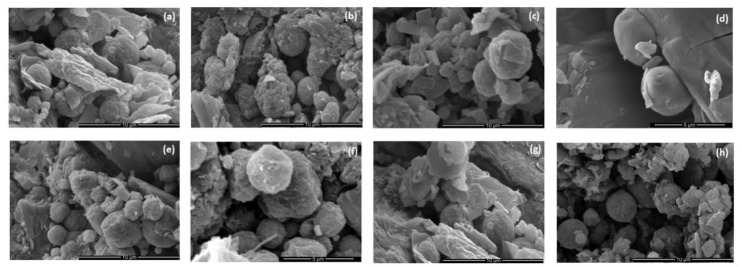
SEM pictures of ODMT cross-sections: (**a**) formulation F3, (**b**) formulation F4, (**c**) formulation F7 under magnification 10,000×, (**d**) formulation F8 under magnification 50,000×, (**e**) formulation F11 under magnification 10,000×, (**f**) formulation F12 under magnification 50,000×, (**g**) formulation F15, (**h**) formulation F16 under magnification 10,000×.

Physical parameters of prepared tablets might be described as relatively good as the balance between the mechanical properties (sufficient hardness, friability <1%) and the quick disintegration time was captured. The obtained ODMT were hard enough that they did not crush while handling, and simultaneously, the formulations were characterized by the desired rapid disintegration time (below 30 s) (Table 4). The weight and thickness uniformity of ODMT is essential as it impacts dosing accuracy. The average masses of obtained formulations had values from 12.5 mg to 14.1 mg. Thickness of obtained ODMT was in the range from 1.80 mm to 2.01 mm. The optimal mechanical characteristics and rapid dissolution time are key aspects in orodispersible formulations depending on the conditions applied during the process. Appropriate tensile force is particularly important while tableting microparticles. Too high a value of tensile force could result in cracking microparticles, which has an undesirable effect in the case of taste masking. No microparticles crushing occurred at the applied pressure (Figure 3). The tensile force value 0.9 kN was determined experimentally as optimal. While lower pressure was applied, the tablets were too brittle to handle with, while higher disintegration time was insufficient (>30 s). Friability (in every formulation < 1%) and hardness tests have proven that obtained ODMTs were characterized by mechanical properties adequate enough so as not to be damaged during the manufacturing process or packing. However, the hardness of tablets prepared with microparticles utilization was smaller in comparison to placebo or formulations with pure RUP. RUP loading was in the range from 0.4 to 0.5 mg—the lowest values were marked for F8, F12, F16 and they did not meet pharmacopoeial requirements (<85%) [25].

Disintegration time tests, conducted under conditions imitating those prevailing in the oral cavity (2–7 mL) are recommended [50,51,52,53], therefore tests in vivo with healthy volunteers, on petri dishes and using a texture analyzer were utilized. Regardless of the method, disintegration time of all ODMT formulations was below 30 s, and most formulations disintegrated even below 15 s. The longest disintegration time was recorded for F13, F14, F15, F16—19–24 s. In all tablets, wetting time below 30 s was noted.

**Figure 4:**
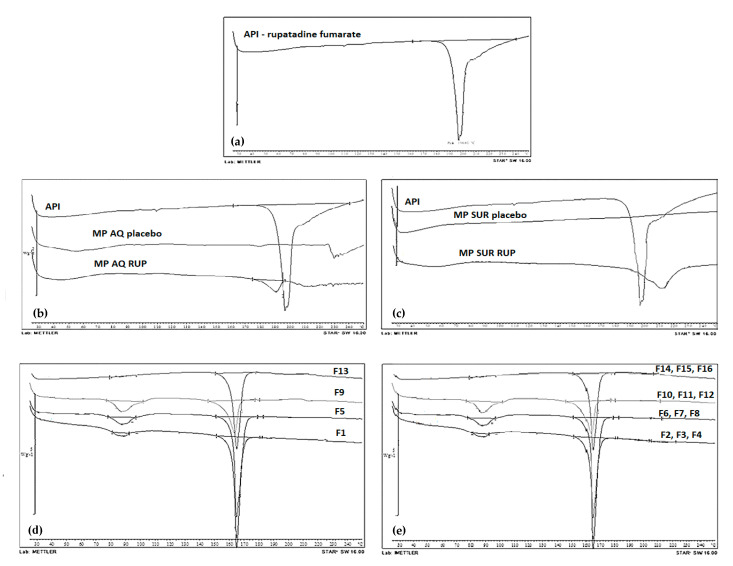
DSC thermograms of RUP - rupatadine fumarate (**a**) microparticles placebo obtained with Aquacoat^®^ or Surelease^®^ (MP AQ placebo MP SUR placebo) and with RUP (MP AQ RUP MP SUR RUP) (**b,c**) ODMT placebo (F1, F5, F9, F13) (**d**) ODMT with pure RUP (F2, F6, F10, F14) and with RUP enclosed in microparticles (F3, F4, F7, F8, F11, F12, F15, F16) (**e**).

An appropriate selection of pharmaceutical excipients is a key issue in creating drug dosage forms, as the excipients might affect physicochemical properties of API. Differential scanning calorimetry (DSC) is one of the analytical techniques frequently applied to determine drug physical properties, as well as to investigate potential incompatibilities with other components. The procedure provides detailed information about the presence of impurities and energetic properties of substances pointing to the differences in the heat flow generated or absorbed by the sample. To evaluate possible interactions, RUP raw material (API), microparticles placebo (MP AQ placebo, MP SUR placebo), microparticles (MP AQ RUP, MP SUR RUP), ODMT placebo (F1, F5, F9, F13), ODMT with pure RUP (F2, F6, F10, F14) and ODMT with RUP enclosed in SUR MP (F3, F7, F11, F15) and AQ MP (F4, F8, F12, F16) (Figure 4) were assessed. RUP chemical nomenclature is 8-chloro-6,11-dihydro-11-[1-[(5-methyl-3-pyridyl)methyl]-4- piperidylidene]-5H-benzo[5,6]cyclohepta[1,2-b]pyridine fumarate [54]. Its melting point should range from 194 to 201 °C. In the literature, there are no polymorphic forms reported for RUP [54,55]. The thermogram of pure RUP presents endothermic event at 196.44 °C characterized by a sharp pick, corresponding to its melting point. Sample decomposition after melting can be observed. Exothermic event transition is shown at 210.35 °C. Both melting and decomposition was noted in a constricted range of temperatures. No additional thermal events connected with decomposition or loss of surface water were observed. Thermograms of microparticles show that there are no thermal events for AQ MP and SUR MP placebo, which indicates that used aqueous dispersion of EC are in an amorphous state. Converting RUP into microparticle form by the spray drying did not significantly change solid state nature of the drug; however, some changes in its melting point occurred—in case of AQ MP RUP the peak has been shifted to 190.67 °C and for SUR MP RUP to 210.1 °C, which indicates that its melting point decreased about 6 °C or increased about 14 °C in microparticle samples, respectively. This is probably due to the fact that excipients used can slightly change physicochemical properties of API during spray drying. In all ready-made co-processed mixtures, the main ingredient is D-mannitol, whose melting point ranges from 155 °C to 165 °C, what was confirmed in the obtained thermogram. There is also a peak in 87 °C of magnesium stearate. No changes in the position of melting peaks and their specific heats were observed in the thermograms of ODMT. There are no further peaks of RUP as API dissolves at the mannitol melting point. No distinct interactions between RUP and used excipients were observed.

### 3.2. Taste-Masking Efficiency Evaluation

Evaluation of taste masking effectiveness is a significant issue, as there are no pharmacopoeial and universal methods to assess the taste. To determine taste masking degree, in vivo (human taste panel) and various in vitro methods (e-tongue, drug release) can be utilized. Human taste panel is the most frequently used strategy of taste evaluation as it is widely available; however, it presents a certain challenge. There are high variances in human taste receptors expression and differences in taste perception (e.g., smoking or taking medicines have an impact). As well, children’s participation in such a study is considered to be unethical, in turn the results obtained in adults are difficult to extrapolate to the entire population due to the different perception of taste sensations. Nevertheless, the predominant approach of assessing the taste of raw medicines and drug dosage forms is by human volunteers. An alternative approach is electronic tongues utilization. It is an analytical gustatory tool for automatic analysis of drug taste. Its essential element is the sensor array composed of chemical sensors with various selectivity. Potentiometric signals recorded in the tested sample do not provide direct information about the composition of the sample, but create its specific digital chemical image, whose interpretation allows to identify a sample or the content of its individual components, including those responsible for generating the taste. Evaluation of bitter taste can also be correlated to the drug release rate. It seems to be the simplest way to determine taste-masking efficacy based mainly on the quantification of drug concentration [56,57,58,59].

#### 3.2.1. In Vivo Taste Evaluation

Initially, six selected healthy volunteers assessed ODMT formulations containing microparticles (F3, F4, F7, F8, F11, F12, F15, F16) as non-bitter or slightly bitter in comparison to those with pure RUP (F2, F6, F10, F14), which were determined as moderately or very bitter (Table 5). It should be also mentioned that mannitol—the main component of obtained ODMT—besides being a sweetening agent, while dissolving in the mouth maintains an impression of cooling, which has a favorable effect on taste sensation during the application [31].

#### 3.2.2. In Vitro RUP Release

**Figure 5:**
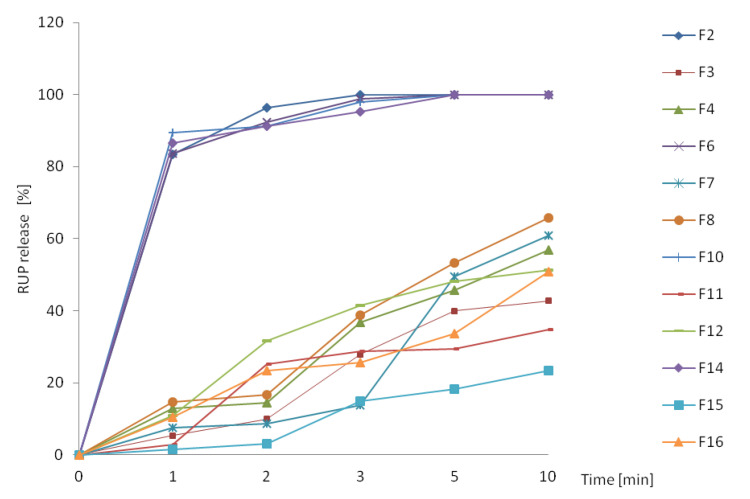
RUP release from designed ODMT performed in paddle apparatus.

Taste masking was also evaluated by RUP release from obtained formulations. Slowing the release of a drug is associated with better efficacy of masking the taste. ODMT made with microparticles (F3, F4, F7, F8, F11, F12, F15, F16) released RUP significantly slower compared to ODMT with pure RUP (F2, F6, F10, F14), where immediate release of RUP occurred (Figure 5). After one minute of dissolution test, maximum 15% of RUP was released, which indicates satisfactory taste masking effect considering very quick disintegration time (about 20 s) and short residence time in the oral cavity.

#### 3.2.3. Electronic Tongue

**Figure 6:**
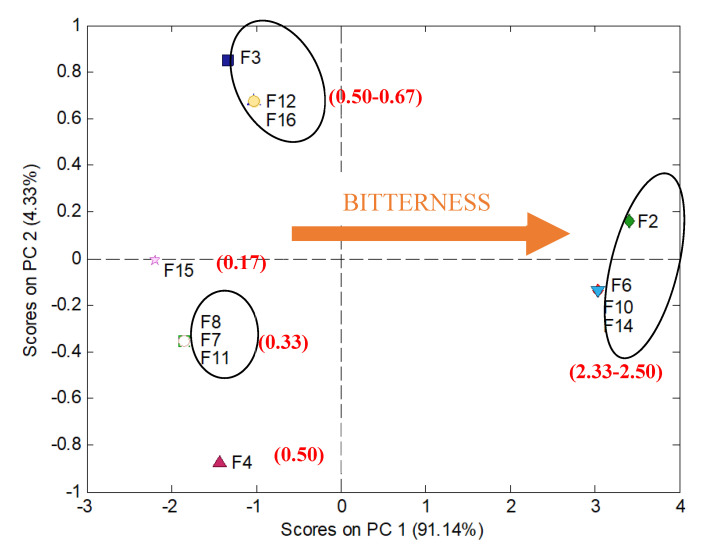
PC1, PCA of human panel responses showing similarity of sensed bitterness for the studied minitablets. Values in brackets show mean values of bitterness score calculated from Table 5.

To investigate in detail taste masking efficiency for the studied formulations, human panel responses were processed by means of a multivariate technique—PCA. This data analysis method helps to find the most significant information hidden in the multidimensional data structure. As a result, a score plot in principal components (PC) coordinates is obtained, which shows clusters of samples based on their similarity. The more similar multidimensional characteristics are, the closer are the objects in the PC1-PC2 space. PCA scores plot (Figure 6) presents PCA processed data of human panel responses shown in Table 5.

The formulations form various clusters according to the sensed bitterness. The most bitter formulations: F2, F6, F10, F14, that reached mean score higher than 2, are placed close to each other and are characterized by high value of PC1. F6, F10, and F14 were evaluated identically by all volunteers, therefore they are overlapping, having the same coordinates PC1-PC2. Only one volunteer (a) estimated F2 as very bitter in contrast to F6, F10, F14 scored by him/her as moderately bitter, therefore F2 is similar to F6, F10, F14 PC1-PC2 scores, but not the same. ODMT F15 are placed in the highest distance from F2, F6, F10, and F14 cluster, having the lowest value of PC1, because they were estimated as not bitter by 5 out of 6 volunteers (the lowest mean value of bitterness). All remaining formulations were scored as very slightly bitter (mean values of bitterness from 0.33 to 0.67), and accordingly, they exhibit moderate PC1 values. All these observations perfectly match the dissolution tests (Figure 5), where formulations F2, F6, F10, F14 show high dynamics of RUP release, whereas the slowest release in the first two minutes is observed in the case of F15 minitablets.

**Figure 7:**
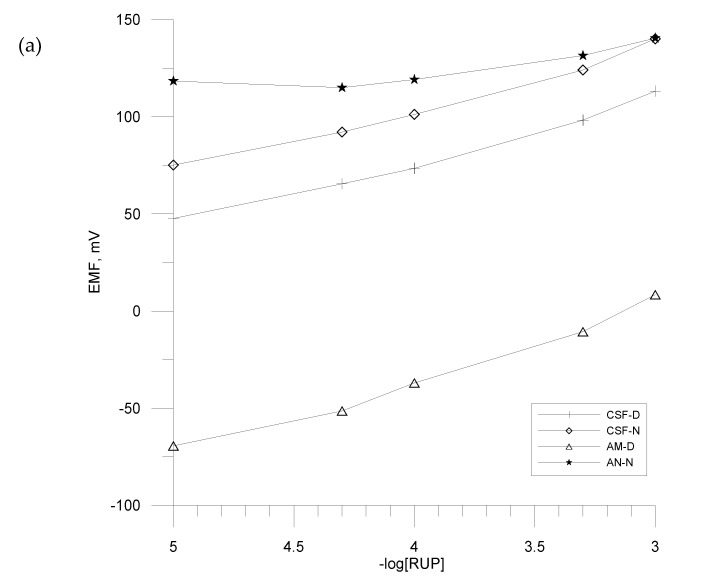

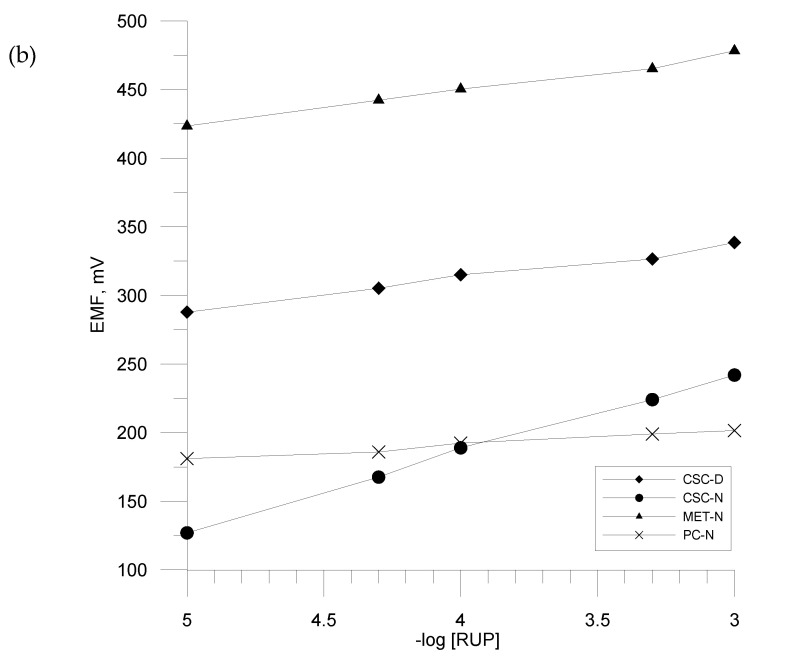
PC2, calibration curves of ion-selective electrodes with CSF, AM, AN (**a**) and CSC, MET, PC (**b**) in 10^−5^–10^−3^ mol L^−1^ RUP solutions.

Before the measurements of pharmaceutical formulations, an important stage of research was optimization of the sensor array. For this purpose, calibration curves of electrodes towards RUP were determined. As it results from Figure 7, for all electrodes containing various active substances and plasticizers in the membrane, different sensitivity towards RUP was achieved. Sensitivity ranged from about 10 mV decade^−1^ to about 51 mV decade^−1^ of 10^−5^–10^−3^ mol L^−1^.

The electrodes based on KTFPB (CSF-D, CSF-N) displayed very similar calibration curves, with mean sensitivity 29.2 ± 4.3–32.4 ± 0.89 mV decade^−1^ in the 10^−5^–10^−3^ mol L^−1^ linear range, good mean correlation coefficient R^2^ = 0.9944 (n = 3). The electrodes containing KTpCPB reveal visible differences between each other; the sensor‘s membrane plasticized with DOS (CSC-D) showed lower mean sensitivity 23.1 ± 4.4 mV decade^−1^ (R^2^ = 0.9983) than electrode with o-NPOE. This electrode exhibited linear range with slope close to the near Nernstian 51.5 ± 4.0 mV decade^−1^ and good correlation coefficient R^2^ = 0.9993. Slightly lower sensitivity exhibited the electrode with amine ionophore, mean response 41.5 ± 4.6 mV decade^−1^. Moreover, the lowest slope of characteristics was obtained for electrodes prepared with ammonium and pyridinium ion exchangers. The slope coefficient of linear range of characteristic amounts 10 mV decade^−1^ for PC-N electrode and 6 mV decade^−1^ for AN-N electrode. The sensor based on metrian (MET-N) showed a similar response to electrodes CSF. All electrodes possessed lower or higher sensitivity to ionic RUP molecules (carboxyl groups, protonated nitrogen atom) as a result of the interaction of RUP with the active components of the polymeric membrane. Concluding, the prepared electrodes of electronic tongue sensor array exhibited satisfactory sensitivity towards studied API. According to our previous studies [17,60,61], such sensors are also cross-sensitive, responding to various excipients, which is a necessary condition for electronic tongue study.

#### 3.2.4. Electronic Tongue—Taste Evaluation

**Figure 8:**
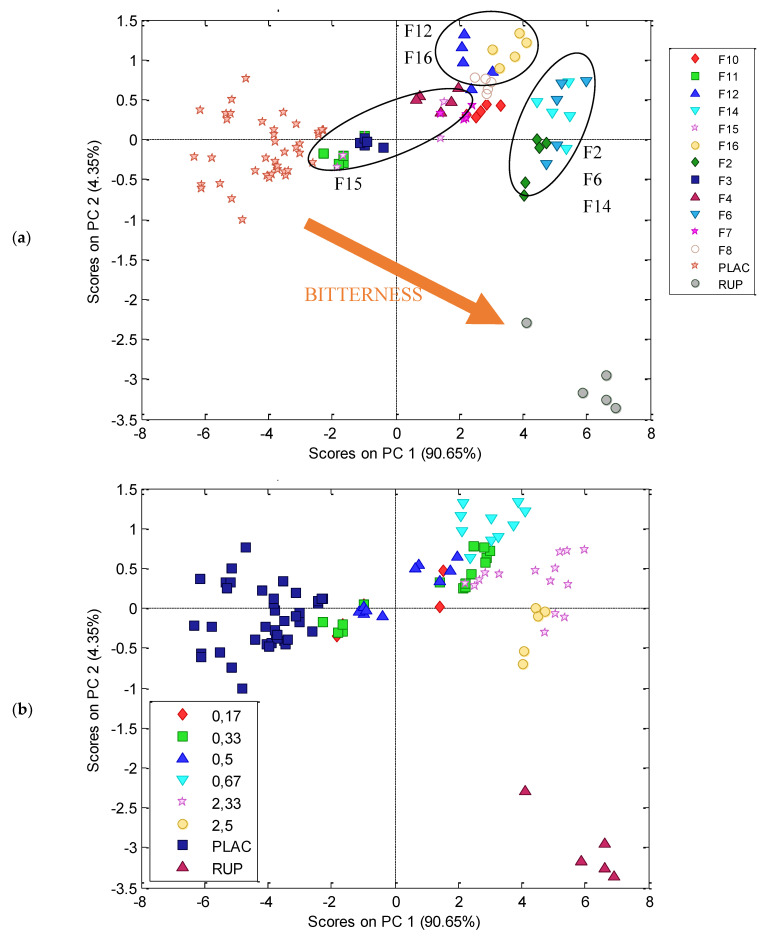
PC3, PCA score plot of electronic tongue responses for all studied formulations (F2-F16), respective placebos (PLAC) and pure RUP. On both plots the same object are presented, therefore they are in the same configuration, but the symbols are given according to: (**a**) formulation type; (**b**) mean values of bitterness score calculated from Table 5.

The prepared sensor array was applied to check taste masking efficiency of all prepared ODMT. The procedure of measurements are presented in the experimental section. According to it, the responses of every sensor was given as ΔEMF in a function of time. Taste evaluation was performed for signals recorded after two minutes of release and resulting PCA score plot is presented in Figure 8.

**Figure 9:**
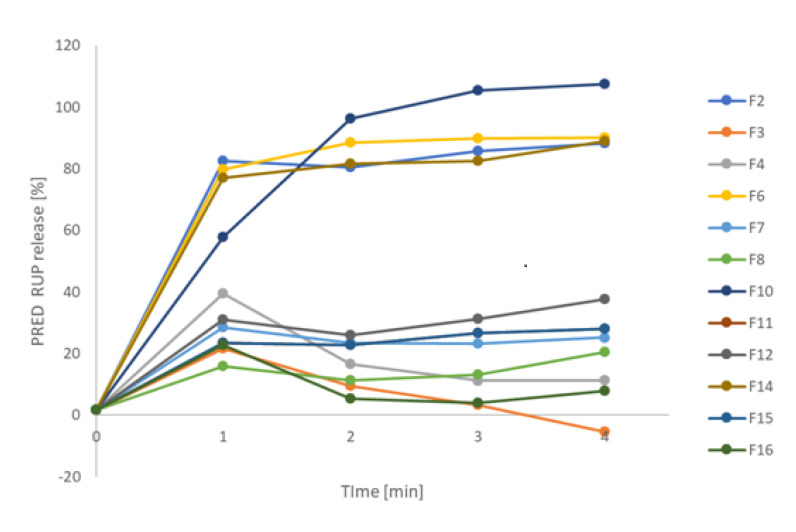
Electronic tongue prediction of RUP release (the results for independent test set).

All placebos formed a distinct cluster; they are grouped together even though their composition is different. On the opposite side of the plot, pure RUP samples are observed. All studied formulations take place between placebos and API, showing moderate taste between the two, which is correct and was expected. The clusters of formulations are partially overlapping, however similarity between electronic tongue study and human panel evaluation can be noticed. There is a cluster formed by F2, F6, and F14 ODMT in the closest distance to pure RUP, therefore their bitterness is most similar to pure API. Moreover, in proximity of this cluster, F10 can be seen. These four formulations showed the highest bitterness according to human panel (Figure 6) and highest dynamic of RUP release (Figure 8). The closest to RUP samples are F2 minitablets, which were evaluated as the most bitter, having a mean value of bitterness score equal to 2.5. The formulation that was the closest to placebo (not bitter) was F15, having the lowest bitterness score (0.17) and this fact also correlates well with human panel results and dissolution study. However, cluster of F15 is not distinct, it is spread out between other formulations, overlapping, e.g., F3 and F11 minitablets. Generally, samples having similar taste sensed by the human panel are considered similar also in terms of electronic tongue response, e.g., F8 is close to F7, and F12 is close to F16. The correlation is not perfect—the most surprising is the position of F11, in high distance from F7 and F8 minitablets. Nevertheless, the results of electronic tongue study reveal highest efficiency of taste masking for formulation F15 and lowest for formulations F2, F6, F10, and F14, which was confirmed by dissolution tests (Figure 5 and Figure 9) and human panel results.

#### 3.2.5. Electronic Tongue—Prediction of Dissolution Study

Signals of electrodes forming sensor array of electronic tongue were recorded during 10 min of formulation release. These outputs are related to RUP release, because the sensors are sensitive towards this API; however, they are also strongly influenced by increasing concentration of excipients, which are also released, due to cross-sensitivity of sensors. Therefore, to extract information of RUP release from sensor responses, a supervised data analysis technique was applied. First, we attempted to construct a Partial Least Squares (PLS) model. PLS regression is a chemometric procedure combining PCA and multiple regression. This approach leads to the possibility of the prediction of dependent variables from independent variables (measurement data). It is performed by transforming the obtained results into so-called latent components enabling the calculation of the dependent variables [62]. We applied this data analysis technique for various applications of the electronic tongue system [63,64,65]. In this paper, electronic tongue signals in appropriate time points were used for PLS modeling. All obtained data were divided into the training and test set. Target matrix was constructed based on %RUP release values that were determined by a standard dissolution test. For establishing a PLS model, a training set of data was applied aiming to find a correlation between the sensor array signals in an appropriate time point and % of RUP released in a respective formulation in a respective time point. When model was ready, the electronic tongue system was capable of predicting of amount of RUP that was released from the respective formulation based on electrodes’ signals. The values of the RUP release were obtained for all studied formulations for a few time points. The resulting dissolution curves for independent test set data are presented in Figure 9. It must be underlined that what is predicted by electronic tongue system values are estimates, they do not provide accurate values of RUP release. The most evident example of that fact are negative values of RUP release for F3 minitablets. However, the outputs of PLS model show general tendencies discerning the studied formulations according to release dynamics. Two groups of dissolution curves can be observed. According to electronic tongue signals, four kinds of ODMT: F2, F6, F10, and F14, released RUP very fast, whereas all other formulations were characterized by much slower dynamics of its release. This finding correlates well with the standard dissolution test (Figure 5).

## 4. Conclusions

Minitablets are getting increasingly significant among modern solid oral drug dosage forms, enabling the application of medicinal substances for patients of all ages by dose multiplying. Due to their small size they are easy to administer and swallow. Minitablets in orodispersible form improve patient compliance in relation to rapid disintegration in the oral cavity within seconds in the presence of the saliva without requirement of drinking water. Orodispersible minitablets (ODMT) with rupatadine fumarate were successfully prepared by direct compression of commercially available ready to use blends and ethylcellulose microparticles. Designed ODMT were characterized by beneficial physicochemical parameters. The presented study also indicates that MP made of etyhylcellulose with rupatadine fumarare might be efficiently utilized in preparation of ODMT by direct compression technique. The evaluation of taste masking level undertaken by three alternative approaches (e-tongue assessment, volunteers, and the drug release) established that designed ODMT are efficient taste–masking carriers of rupatadine fumarate and are supposed to be a promising alternative for traditional tablets.

## Figures and Tables

**Table 1:** Composition of orodispersible minitablets (ODMT) formulations.

Ingredient [%]	Formulation															
	F1	F2	F3	F4	F5	F6	F7	F8	F9	F10	F11	F12	F13	F14	F15	F16
**RUP**	-	3.5			-	3.5			-	3.5			-	3.5		
**SUR MP RUP** *[corresponding to 0.5 mg RUP per one tablet]*	-		7.15		-	-	7.15		-	-	7.15		-	-	7.15	
**AQ MP RUP** *[corresponding to 0.5 mg RUP]*	-			8.65	-	-		8.65	-	-		8.65	-	-		8.65
**Parteck^®^ ODT**	99	95.5	91.85	90.35	-	-			-	-			-	-		
**SmartEx^®^ QD-50**	-				99	95.5	91.85	90.35	-	-			-	-		
**F-Melt C**	-				-	-			99	95.5	91.85	90.35	-	-		
**Pearlitol^®^ Flash**	-				-	-			-				99	95.5	91.85	90.35
**Magnesium stearate**	1	1	1	1	1	1	1	1	1	1	1	1	1	1	1	1

**Table 2:** Electrodes forming sensor array of the electronic tongue.

Electrode Number no.	Electrode Type	Ionophore (%, *w*/*w*)	Lipophilic Salt (%, *w/w*)	Plasticizer (%, *w/w*)	Polymer (%, *w/w*)
1–2	CSF-D	−	KTFPB (1%)	DOS (66%)	PVC (33%)
3–4	CSF-N	−	KTFPB (1%)	o-NPOE (66%)	PVC (33%)
5–6	CSC-D	−	KTpCPB (3%)	DOS (64%)	PVC (33%)
7–8	CSC-N	−	KTpCPB (3%)	o-NPOE (64%)	PVC (33%)
9–10	AM-D	Amine ionophore I (5%)	−	DOS (68%)	PVC (27%)
11–12	MET-N	METRIAN (4%)	−	o-NPOE (66%)	PVC (30%)
13–14	PC-N	−	DDPC (3%)	o-NPOE (64%)	PVC (33%)
15–16	AN-N	−	TDMAC (4%)	o-NPOE (66%)	PVC (30%)

**Table 3:** Characteristics of tableting blends.

Powder Mixture	Density [g/mL]		Flow Properties	
	Bulk	Tapped	Hausner’s Ratio	Carr’s Index [%]
**F1**	0.58	0.72	20.45	1.26
**F2**	0.58	0.71	20.45	1.26
**F3**	0.56	0.70	20.40	1.27
**F4**	0.57	0.68	20.20	1.24
**F5**	0.51	0.65	15.38	1.28
**F6**	0.52	0.65	15.38	1.28
**F7**	0.50	0.63	15.39	1.29
**F8**	0.49	0.62	15.25	1.30
**F9**	0.56	0.67	13.25	1.16
**F10**	0.56	0.66	13.24	1.16
**F11**	0.54	0.62	13.23	1.17
**F12**	0.52	0.61	13.22	1.15
**F13**	0.48	0.57	13.10	1.15
**F14**	0.48	0.58	13.10	1.15
**F15**	0.45	0.54	13.05	1.19
**F16**	0.44	0.53	13.13	1.16

**Table 4:** Physicochemical characteristics of prepared ODMT.

Parameter	F1	F2	F3	F4	F5	F6	F7	F8	F9	F10	F11	F12	F13	F14	F15	F16
Formulation																
Weight [mg] *	13.4 ± 0.2	13.7 ± 0.3	14.0 ± 0.7	13.6 ± 0.5	14.1 ± 0.3	12.6 ± 0.9	12.9 ± 0.6	12.5 ± 0.7	13.8 ± 0.3	13.4 ± 0.2	13.5 ± 0.4	13.4 ± 0.5	14.1 ± 0.2	13.4 ± 0.5	13.9 ± 0.3	13.70 ± 0.4
Thickness * [mm]	2.01 ± 0.1	2.01 ± 0.1	1.96 ± 0.2	1.94 ± 0.3	1.94 ± 0.1	1.96 ± 0.2	1.82 ± 0.2	1.80 ± 0.3	2.0 ± 0.1	1.98 ± 0.1	1.97 ± 0.3	1.95 ± 0.3	1.97 ± 0.1	1.95 ± 0.1	1.95 ± 0.3	1.93 ± 0.4
Hardness [N] ** *(by hardness tester)*	15.4 ± 3.4	14.4 ± 2.4	8.40 ± 4.5	8.20 ± 4.7	16.8 ± 2.2	16.2 ± 2.5	8.1 ± 2.1	7.8 ± 2.3	15.9 ± 2.5	15.1 ± 2.1	8.1 ± 2.7	7.8 ± 2.9	16.1 ± 1.2	15.8 ± 2.1	8.1 ± 3.7	7.5 ± 4.2
Hardness [N] ** *(by texture analyzer)*	15.1 ± 3.9	14.6 ± 2.3	8.1 ± 4.0	8.0 ± 4.4	16.7 ± 2.2	16.5 ± 2.4	8.1 ± 1.5	7.8 ± 2.5	16.0 ± 2.2	15.0 ± 2.0	8.2 ± 2.5	8.0 ± 2.8	16.1 ± 1.1	15.9 ± 2.3	8.0 ± 3.5	7.6 ± 4.1
**Friability [%]**	0.1	0.1	0.1	0.2	0.1	0.45	0.1	0.1	0.1	0.1	0.1	0.3	0.1	0.1	0.1	0.2
Drug content *** [mg]	-	0.47 ± 0.1	0.48 ± 0.3	0.43 ± 0.4	-	0.45 ± 0.1	0.43 ± 0.2	0.42 ± 0.3	-	0.47 ± 0.1	0.45 ± 0.3	0.41 ± 0.4	-	0.49 ± 0.1	0.5 ± 0.1	0.40 ± 0.2
**% of declared dose**	-	94	96	86	-	90	86	84	-	94	90	82	-	98	100	0.80

*—the test was performed for 20 tablets [25]; **—the test was performed for 10 tablets in triplicate; ***—the test was performed for 10 tablets [25].

**Table 5:** Sensory evaluation of designed ODMT formulations, estimated as follows: 0—no bitterness, 1—slightly bitterness, 2—moderately bitterness, 3—significantly bitterness.

Volunteer	Score											
	F2	F3	F4	F6	F7	F8	F10	F11	F12	F14	F15	F16
**A**	3	0	0	2	0	0	2	0	1	2	0	1
**B**	3	1	1	3	1	1	3	1	1	3	1	1
**C**	2	1	0	2	0	0	2	0	1	2	0	1
**D**	2	0	1	2	0	0	2	0	0	2	0	0
**E**	3	0	1	3	1	1	3	1	1	3	0	1
**F**	2	1	0	2	0	0	2	0	0	2	0	0
